# Female breast cancer survival in Qidong, China, 1972–2011: a population-based study

**DOI:** 10.1186/1471-2407-14-318

**Published:** 2014-05-06

**Authors:** Jian Zhu, Jian-Guo Chen, Yong-Sheng Chen, Yong-Hui Zhang, Lu-Lu Ding, Tao-Yang Chen

**Affiliations:** 1 Qidong Liver Cancer Institute, Qidong Cancer Registry, Qidong People’s Hospital, Qidong 226200, China; 2 Nantong University Tumor Hospital/Institute, School of Public Health, Nantong 226361, China

**Keywords:** Breast cancer, Cancer registration, Observed survival, Relative survival, Qidong

## Abstract

**Background:**

Based on data from the population-based Qidong Cancer Registry, we report a survival analysis for female breast cancer patients diagnosed during 1972**–**2011 in order to assess the long-term trends for the prognosis of this cancer.

**Methods:**

The last follow-up for survival status of the 3,398 registered female breast cancer cases was April, 2012. Cumulative observed survival (OS) and relative survival (RS) rates were calculated using Hakulinen’s method performed by the SURV3.01 Software developed at the Finnish Cancer Registry.

**Results:**

The one-, three-, five-, ten-, fifteen-, twenty-, thirty-, and forty- year OS rates were 83.61%, 67.53%, 58.75%, 48.56%, 42.57%, 38.30%, 29.19%, 19.35%; and the RS rates were 84.76%, 70.45%, 63.12%, 56.81%, 55.26%, 56.36%, 62.59%, 84.00%, respectively. Five-year RS rates of age groups 15**–**34, 35**–**44, 45**–**54, 55**–**64, 65**–**74, and 75+ were 60.17%, 68.27%, 67.79%, 56.03%, 55.50%, and 57.28%; 10-year RS rates were 54.16%, 59.59%, 61.34%, 47.78%, 51.30%, and 59.28%, respectively. There were statistical differences among the age groups (RS: χ^2^ = 152.15, *P* = 0.000). Remarkable improvement could be seen for the 5-year RS rates from 52.08% in 1972 to 69.26% in 2003**–**2007, and the 10-year RS rates from 43.16% in 1972 to 60.85% in 1998**–**2002, respectively.

**Conclusions:**

Survival outcomes from Qidong registered cases with breast cancer have shown gradual progress during the past 40 years. The disparities between survival rates of this area and developed countries are getting narrower, but there is still great need for improving survival in Qidong.

## Background

This study was based on the data of the Qidong Cancer Registry (QCR), a population-based cancer registry, which was established in 1972 and is administered by the Qidong Liver Cancer Institute (QDLCI). The QCR covers Qidong City, Jiangsu Province, China with an area of 1208 km^2^, and a population of 1.12 M at the end of 2011. Geographically, Qidong is located at the mouth of the Yangtze River (*Chang Jiang*), by the Yellow Sea (*Huang Hai*). The QCR is one of the longest standing cancer registries in China. In 1974, the QDLCI established an all-death cause registration system (as an official Vital Statistics source) in the same coverage area for disease monitoring.

Breast cancer is now the most common cancer in females all over the world [1,2]. With the development of the economy and the improvement of living standards, the incidence of breast cancer is increasing year by year in China [3]. In Qidong, breast cancer has been the fourth most common cancer in females over the past 40 years, with an aggregate crude incidence rate of 14.94 per 100 000, accounting for 9.95% of all cancer sites combined in women. The annual percentage change (APC) of the world age-standardized incidence rate between 1972 and 2011 was 1.31%, with a rapid increase during the latter stages of the past four decades [4]. Thus, further research on breast cancer etiology, prevention and treatment hence is necessary.

This study presents current survival data and their trends during the period of 1972–2011. The data collected over this long-term period and the results accurately derived from them reflect a profile for the prevalence and management of female breast cancer in Qidong. This information on survival provides clinicians and researchers with opportunities to explore breast cancer trends, to measure progress against breast cancer, and to examine implications for breast cancer control in the context of associations with risk factors, prevention interventions, and the dissemination of advances in treatment.

## Methods

The data in this study are from the QCR, which at its founding was designated as the cancer registration repository by the local health authority with compulsory reporting by health care workers. Several years later cancer registration was mandated to be compulsory by the provincial health authority. Now this cancer registry is a member of the national monitoring program (or the National Cancer Registration Network) [5,6] of the National Central Cancer Registries of China supported by the Ministry of Finance and the Ministry of Health of China: Tumor Follow-up Registration Programs (MF2008-293, 2009–193, and 2010–90).

The Qidong All-death Cause Registration System has been an official vital statistics source approved by the Ministry of Health of China since 1974 [7], with death from any cause reported by death certification notifications (DCN). Meeting International Agency for Research on Cancer/International Association of Cancer Registries (IARC/IACR) standards for quality, completeness, timeliness and unresolved duplicate records, the QCR is a member of the IACR. QCR data have been included in “Cancer Incidence in Five Continents” (CI5) [8,9] and other publications [10-13]. The data reliability has earned recognition in domestic and international studies [10,12,14]. For the use of these cancer statistics, there is no further need for approval by an Institutional Review Board and no need for informed consent from any of the patients involved.

The QCR uses both active and passive methods for cancer data collection. All data files received from lower-level registries and all other hospitals are checked with cancer report lists and DCNs in order to track down missing cases and to exclude duplicate registrations. At present, there are 12 town-level registries, each with one full-time or part-time health worker doing the registration and collection of DCNs. All new cancer patients in the catchment area are therefore registered, checked, and then reported to the QCR. When the patient dies, whether at home or in hospital, the registration official adds the date of death to the record, and reports it again, together with a DCN card. If the registry personnel receive the death notification first, the patient’s medical records are reviewed or a home visit is carried out to obtain further information. Hence, the collection of high-quality follow-up data has been possible in Qidong [10,11].

According to Union for International Cancer Control (UICC), breast cancer refers to the malignant tumor originating in the mammary gland epithelial tissue. In QCR, cancer cases were classified and coded according to the International Classification of Diseases, the 10^th^ revision (ICD-10), and the International Classification of Diseases for Oncology 3^rd^ edition [15] as well. This study included all patients with female breast cancer reported to the QCR from 1972 to 2011; codes of ICD-10 C50.0-C50.9 and topography codes C50.0-C50.9 of ICD-O-3 with behaviour codes 3 were included. Excluded were all death certificate-only, autopsy, and individual cases with malignant secondary (metastatic to the breast), benign, in situ and uncertain or unknown diagnosis.

A total of 3,452 cases with breast cancer were registered from January 1, 1972 to December 31, 2011. Fifty-four (1.56%) male breast cancer cases were excluded, hence 3,398 female breast cancers were included for analysis. The deadline for the last follow-up for survival status was April, 2012. The proportion of cases with morphological verification was 93.11% (3,164/3,398), with no breast cancer cases recognized by death certificate only. The survival duration of each case was determined as the time difference from the date of initial diagnosis to the date of death due to breast cancer (1,687/3,398 = 49.65%), date of death due to other diseases (3/3,398 = 0.09%), date of loss to follow-up (381/3,398 = 11.21%), and the closing date for those still alive (1,327/3,398 = 39.05%).

Cumulative observed survival (OS) and relative survival (RS) rates were calculated. RS was defined as the ratio of the OS rate to the expected rate, which was estimated from the general gender and calendar period-specific life tables for Qidong residents according to Hakulinen’s method [16]. The survival experience of patients were adjusted for normal life expectancy of the general population of the same age, which makes RS rate an estimate of the chance of surviving the effects of cancer [17]. The OS and RS rates were computed within six age groups (15–34, 35–44, 45–54, 55–64, 65–74, 75+) and nine calendar periods (1972, 1973–1977, 1978–1982, 1983–1987, 1988–1992, 1993–1997, 1998–2002, 2003–2007, 2008–2011), using Hakulinen’s method performed by the SURV3.01 software developed at the Finnish Cancer Registry [18]. Survival trends could be shown through comparing different calendar periods. Statistical tests are included in this software. Relative rates among different age groups were tested using H_0_-H_2_ equal *vs*. unequal hazards tests.

## Results

### Observed survival and relative survival rates

**Figure 1:**
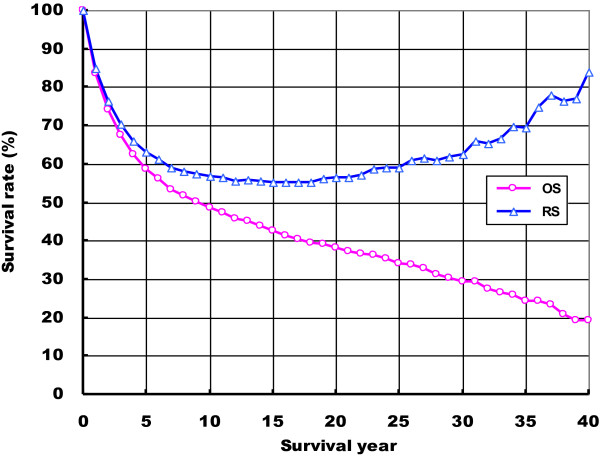
**Trends for the observed survival (OS) and relative survival (RS) rates of female breast cancer in Qidong, China, 1972–2011.** (−Ο−) OS; (−Δ−) RS.

Table 1 shows OS and RS rates by the survival year. The one-, three-, five-, ten-, fifteen, twenty, thirty, and forty-year OS rates of female breast cancer in Qidong during 1972–2011 were 83.61%, 67.53%, 58.75%, 48.56%, 42.57%, 38.30%, 29.19%, and 19.35%, while the RS rates were 84.76%, 70.45%, 63.12%, 56.81%, 55.26%, 56.36%, 62.59%, and 84.00%, respectively. Figure 1 indicates OS and RS trends of female breast cancer in Qidong during 1972–2011 by the survival year. Significant disparities between OS and RS rates could be found in female breast cancer patients surviving over 10 years. For survivors more than 20 years after diagnosis, RS rates increased with the survival year, implying that these women with breast cancer have a relatively higher survival chance compared to the general population with the same demographical characteristics.

**Table 1:** Observed survival (OS) and relative survival (RS) rates of female breast cancer in Qidong, 1972–2011 (%)

**Survival year**	**OS**	2*SE_**OS**_	**RS**	2*SE_**RS**_
**1**	**83.61**	**1.29**	**84.76**	**1.31**
2	74.12	1.55	76.22	1.59
**3**	**67.53**	**1.68**	**70.45**	**1.75**
4	62.41	1.76	66.06	1.87
**5**	**58.75**	**1.81**	**63.12**	**1.95**
6	56.04	1.85	61.16	2.02
7	53.20	1.89	59.00	2.09
8	51.58	1.91	58.18	2.16
9	50.07	1.93	57.49	2.22
**10**	**48.56**	**1.96**	**56.81**	**2.29**
11	47.35	1.98	56.49	2.36
12	45.71	2.01	55.64	2.44
13	45.06	2.02	55.98	2.51
14	43.75	2.05	55.53	2.60
**15**	**42.57**	**2.08**	**55.26**	**2.70**
16	41.48	2.10	55.11	2.80
17	40.49	2.13	55.10	2.90
18	39.53	2.16	55.16	3.01
19	39.26	2.17	56.22	3.10
**20**	**38.30**	**2.20**	**56.36**	**3.23**
21	37.35	2.23	56.58	3.38
22	36.63	2.26	57.23	3.54
23	36.38	2.28	58.69	3.67
24	35.27	2.34	58.86	3.90
25	34.20	2.40	59.15	4.16
26	33.85	2.43	60.80	4.36
27	32.89	2.51	61.49	4.69
28	31.18	2.65	60.85	5.18
29	30.24	2.73	61.77	5.59
**30**	**29.19**	**2.83**	**62.59**	**6.07**
31	29.19	2.83	65.82	6.39
32	27.54	3.03	65.42	7.20
33	26.41	3.18	66.47	8.00
34	25.95	3.26	69.66	8.74
35	24.16	3.63	69.47	10.43
36	24.16	3.63	74.70	11.22
37	23.23	3.94	77.82	13.19
38	20.96	4.68	76.35	17.04
39	19.35	5.31	76.90	21.12
**40**	**19.35**	**5.31**	**84.00**	**23.07**

2*SE_*OS*_, Twice standard error of OS; 2*SE_*RS*_, Twice standard error of RS.

### Survival rate by age group

Table 2 shows the 5-, and 10-year OS and RS rates by age group. The 5-year OS rates of age groups 15–34, 35–44, 45–54, 55–64, 65–74, and 75+ were 59.81%, 67.42%, 66.26%, 53.44%, 47.82%, and 30.42%, while the 10-year OS rates were 53.36%, 57.82%, 58.09%, 42.11%, 32.05%, and 14.78%, respectively. The 5-year RS rates were 60.17% 68.27%, 67.79%, 56.03%, 55.50%, and 55.28%, and 10-year RS rates were 54.16%, 59.59%, 61.34%, 47.78%, 51.30%, 59.28%, respectively. The patients at 35–44 or 45–54 years of age experienced higher 5-, and 10-year OS and RS rates. Statistical differences could be found among the different age groups (RS: χ^2^ = 152.15, *P* = 0.000).

**Table 2:** Five and ten year OS and RS rates of female breast cancer by age-group in Qidong, 1972–2011 (%)

**Age group**		**15-34**	**35- 44**	**45-54**	**55-64**	**65-74**	**75+**	**Total**
5-year	OS	59.81	67.42	66.26	53.44	47.82	30.42	58.75
	RS	60.17	68.27	67.79	56.03	55.50	55.28	63.12
10-year	OS	53.36	57.82	58.09	42.11	32.05	14.78	48.56
	RS	54.16	59.59	61.34	47.78	51.30	59.28	56.81

OS, Observed survival; RS, Relative survival.

### Survival rate by period

Table 3 shows the RS rates by survival year and by period. An increasing trend for the RS rates of female breast cancer could be observed through the nine intervals during 1972–2011 period. Remarkable improvement could be seen for the 5-year RS rates of 52.08% in 1972 to that of 69.26% in 2003–2007, and for the 10-year RS rates of 43.16% in 1972 to that of 60.85% in 1998–2002. Little improvement was observed for the 1-year RS rate from 87.91% in 1972 to 90.30% in 2008–2011, and for the 15-year RS from 45.81% in 1972 to 48.22% in 1993–1997.

**Table 3:** Relative survival rate of female breast cancer by period in Qidong, 1972–2011 (%)

**Survival year**	**1972**	**1973-1977**	**1978-1982**	**1983-1987**	**1988-1992**	**1993-1997**	**1998-2002**	**2003-2007**	**2008-2011**
**1**	**87.91**	**85.54**	**83.28**	**84.38**	**85.77**	**77.02**	**82.90**	**85.19**	**90.30**
2	73.74	73.66	72.86	73.98	73.73	69.92	74.30	79.47	85.06
3	64.39	63.54	66.27	68.35	67.92	63.17	68.72	75.89	81.55
4	58.35	58.85	62.71	61.85	62.57	58.51	65.18	73.06	75.71
**5**	**52.08**	**56.30**	**60.43**	**59.98**	**60.32**	**54.62**	**63.11**	**69.26**	**-**
6	52.90	54.16	59.07	58.09	57.16	52.64	61.68	67.44	
7	48.15	51.51	56.24	55.79	54.53	51.15	60.49	65.70	
8	47.05	49.72	52.37	55.24	53.99	50.82	61.16	64.38	
9	44.17	49.89	50.33	52.50	54.08	50.47	61.45	65.27	
**10**	**43.16**	**51.13**	**50.24**	**50.82**	**52.92**	**49.76**	**60.85**	-	
11	43.99	51.93	50.14	50.62	52.41	48.70	60.46		
12	44.93	49.55	51.10	48.86	51.84	48.32	59.38		
13	45.87	49.81	51.02	48.63	52.23	48.97	60.30		
14	44.79	50.03	49.88	48.01	51.93	48.67	61.22		
**15**	**45.81**	**49.04**	**50.97**	**47.83**	**51.25**	**48.22**	-		
16	44.67	46.78	49.83	48.10	52.35	48.82			
17	45.82	46.86	49.85	47.91	52.81	47.47			
18	47.05	45.02	49.28	48.11	53.74	48.77			
19	45.76	46.38	50.60	48.80	54.77	50.05			
**20**	**42.02**	**45.83**	**50.77**	**49.04**	**55.89**	-			
21	43.31	43.18	51.66	49.30	57.65				
22	44.70	41.10	53.21	50.15	59.66				
23	43.43	42.62	54.17	51.62	62.06				
24	42.07	41.20	55.22	52.19	64.92				
25	40.72	42.18	54.83	52.69	-				
26	39.31	44.10	56.66	53.66					
27	37.86	44.40	57.18	55.70					
28	36.23	45.61	55.41	54.98					
29	38.07	44.93	56.99	57.12					
**30**	**32.84**	**46.17**	**58.68**	-					
31	34.71	48.56	61.57						
32	36.29	46.81	62.74						
33	38.08	47.39	63.33						
34	40.38	49.38	67.23						
35	38.06	49.98	**-**						
36	40.61	53.91							
37	43.89	55.24							
38	47.55	50.61							
39	51.98	43.96							
**40**	**56.78**	-							

## Discussion

This study provides survival estimates of female patients diagnosed with breast cancer in Qidong, China from 1972 through 2011. We compare the results available from other countries or areas. For example, reports from Korea, Japan, United States, and Germany have shown better relative survival rates of female breast cancer than observed in Qidong. The 5-year RS rate (63.12% in 1972–2011) of female breast cancer in Qidong was lower than those in Korea (79.6% in 1993–1997, 85.0% in 1998–2002) [19], Japan (84.6% in 1993–1996) [20], United States (89.0% in 1996–2002) [21], Germany (80.6% in 2000–2002) [22], and most of other Asia cities or areas (58.6%-88.2% in 1999) [23], but higher than that of Kampala, Uganda (45.4% in 1993–1997) [24], and Mumbai, India (46.2% in 1992–1994) [25]. As Bradley et al. [26] have reported, there are disparities in cancer survival between subjects enrolled in Medicaid and subjects not enrolled in Medicaid, a health insurance program in the United States. Verdecchia et al. [27] indicated that the most direct way for poorer European countries to improve all-cancer survival would be to get richer; for richer countries investment in health technology is important. Hayat et al. [21] suggested these disparities may reflect variations in the prevalence of risk factors, the use of screening tests for early detection, access to health care services, and/or social and demographic factors. In a comparative study of Stockholm and Singapore women with breast cancer, there appear to be differences in the 5-year OS rates of 72% *vs.* 64%, and 5-year RS rates of 80% *vs*. 70%, respectively. In Qidong, the data highlight an improvement in prognosis over the calendar periods, which were likely influenced by marked economic improvement leading to better medical facilities and management of patients from diagnosis through treatment, as well as improved treatment options [28]. At the molecular level, Guo et al. [29] reported that the 5-year survival in female patients with ERα (estrogen receptor α +) is higher than that with ERα (−) in Chinese women.

But no matter how poor the survival shown in this series, compared to results from other parts of the world or from big cities in China, the data indicate that survival rates for breast cancer patients over this 40 year period in Qidong have been improving. Significant upward trends for the 5-year RS rate were observed, increasing from 52.08% in 1972 to 69.26% in 2003–2007, and the 10-year RS rates from 43.16% in 1972 to 60.85% in 1998–2002. The improvement in survival rate between 1972 and 2011 may be attributed to social and economic development, as well as improvement in early detection, such as by increasing the use of mammography, and therapy [30]. Patients also received better quality care, as shown in a pay-for-performance program for breast cancer care in Taiwan [31]. Last century, however, breast cancer screening programs were almost nonexistent in Qidong. It seems reasonable to assume that many of these patients had no chance to receive early detection for the diagnosis of this cancer, and hence, effective treatment was rarely possible. In recently years, screening as well as substantive improvements in diagnosis and treatment have become possible, through which breast cancers could be detected at earlier stages and perhaps more suitable treatment options could be obtained, in turn resulting in improvements in prognosis.

Our 40-year results indicate that 58.75% (2*SE: 1.81%) of female patients with breast cancer survived 5 years or more, with a 5-year RS rate of 63.12% (2*SE: 1.95%). Statistical differences were noted among the different age groups. Higher 5-, 10-year RS rates were observed from the groups of age 35–54 years in our series, which is consistent with a report from a rural area in China by Chen and his colleagues [32]. Ballard-Barbash et al. [33] in a recent systematic review concludes that physical activity is associated with a reduction in breast cancer–specific mortality as well as all-cause mortality; and there is evidence for a dose–response effect of decreasing mortality risk with increasing activity in roughly half of the 27 reported observational studies. This association may be one of the reasons that younger patients had better survival in our series. Besides a variety of known factors including tumor size, nodal status and grade, histological type, improved treatment modalities and screening, and racial differences [34,35], there may be other factors that affect the prognosis of breast cancer, although their mechanisms are unclear. In a meta-analysis which included 43 studies of women diagnosed with breast cancer between 1963 and 2005, Protani et al. [36] illustrated that women with breast cancer who were obese, had poorer survival than women with breast cancer who were not obese, with a HR of 1.33 (95% *CI*: 1.21-1.47). Some suggest [37] that weight management is a key to controlling prevalent co-morbid conditions in breast cancer patients, although it is currently unknown whether post-diagnosis weight loss can improve prognosis and disease-free survival.

Stage is often considered to be the most important factor determining survival [28]. Hayat et al. [21] reported that the 5-year relative survival for those diagnosed at early stage (stage I) is very good (100% for all races combined), but if metastatic disease is diagnosed (stage IV), survival drops to 21% for all races combined. A study in a Finnish population comparing screening-detected and non-screening-detected breast cancer found that breast cancer detected by mammography screening is an independent prognostic factor in breast cancer that is associated with a more favorable survival rate: a 15-year survival of 86% for patients screening-detected *vs*. 68% for patients diagnosed by other methods [38]. Unfortunately, for our population-based cancer registration series, one limitation of our study was that stage information was available sporadically, a common problem in population-based cancer registries worldwide [39]. This limitation does not hinder the assessment of the general evolution of better survival from breast cancer in this area. We believe, as the literature [35] has suggested, that disparities in access and quality of care may be eliminated by understanding and addressing cultural and economic barriers.

## Conclusions

The survival outcomes in women with breast cancer in Qidong, China have shown gradual progress during the past 40 years. There remain disparities of survival rates of female breast cancer comparing Qidong to more developed regions, yet the gaps are getting narrower, and there is continuing opportunity for improving breast cancer survival in Qidong.

## Abbreviations

APC: Annual percentage change; CI: Confidence interval; CI5: Cancer incidence in five continents; DCN: Death certification notifications; DCO: Death certificate only; ERα: Estrogen receptor α; IACR: International Association of Cancer Registries; IARC: International Agency for Research on Cancer; ICD-10: International Classification of Diseases, the 10th revision; OS: Observed survival rate; QCR: Qidong Cancer Registry; QDLCI: Qidong Liver Cancer Institute; RS: Relative survival rate; 2*SE: Twice standard error; 2*SEOS: Twice standard error of observed survival rate; 2*SERS: Twice standard error of relative survival rate.

## Competing interests

The authors declare that they have no competing interests.

## Authors’ contributions

All authors participated in the collection of the data. ZJ carried out the data analyses and interpretation under supervision of CJG. ZJ wrote the first draft of the manuscript. CJG reviewed the data analyses and edited the draft. All authors participated in the revision of the manuscript and approved the final form.

## Pre-publication history

The pre-publication history for this paper can be accessed here:

http://www.biomedcentral.com/1471-2407/14/318/prepub
